# Development of Antibacterial, Degradable and pH-Responsive Chitosan/Guar Gum/Polyvinyl Alcohol Blended Hydrogels for Wound Dressing

**DOI:** 10.3390/molecules26195937

**Published:** 2021-09-30

**Authors:** Muhammad Umar Aslam Khan, Iqra Iqbal, Mohamed Nainar Mohamed Ansari, Saiful Izwan Abd Razak, Mohsin Ali Raza, Amna Sajjad, Faiza Jabeen, Mohd Riduan Mohamad, Norhana Jusoh

**Affiliations:** 1 BioInspired Device and Tissue Engineering Research Group, School of Biomedical Engineering and Health Sciences, Faculty of Engineering, Universiti Teknologi Malaysia, Skudai 81300, Malaysia; saifulizwan@utm.my (S.I.A.R.); mohd.riduan@utm.my (M.R.M.); norhana@utm.my (N.J.); 2 Institute for Personalized Medicine, School of Biomedical Engineering, Med-X Institute, Shanghai Jiao Tong University, Shanghai 200030, China; 3 National Center for Physics, Nanoscience and Technology Department (NS & TD), Quaid-e-Azam University, Islamabad 44000, Pakistan; 4 Institute of Metallurgy and Materials Engineering, Faculty of Engineering and Technology, University of the Punjab, Lahore 54590, Pakistan; iqbaliqra143@yahoo.com (I.I.); mohsin.ceet@pu.edu.pk (M.A.R.); 5 Institute of Power Engineering, Universiti Tenaga Nasional, Kajang 43000, Malaysia; 6 Centre of Advanced Composite Materials, Faculty of Engineering, Universiti Teknologi Malaysia, Skudai 81300, Malaysia; 7 Department of Zoology, Government College University, Faisalabad 38000, Pakistan; amnasajjad7@yahoo.com; 8 Department of Zoology, University of Education, Lahore 54770, Pakistan; drfaizajabeen@ue.edu

**Keywords:** antibacterial, biopolymers, degradation, drug delivery, pH-sensitive, regenerative medicine, wound dressing

## Abstract

The present research is based on the fabrication preparation of CS/PVA/GG blended hydrogel with nontoxic tetra orthosilicate (TEOS) for sustained paracetamol release. Different TEOS percentages were used because of their nontoxic behavior to study newly designed hydrogels’ crosslinking and physicochemical properties. These hydrogels were characterized using Fourier-transform infrared spectroscopy (FTIR), scanning electron microscopy (SEM), and wetting to determine the functional, surface morphology, hydrophilic, or hydrophobic properties. The swelling analysis in different media, degradation in PBS, and drug release kinetics were conducted to observe their response against corresponding media. The FTIR analysis confirmed the components added and crosslinking between them, and surface morphology confirmed different surface and wetting behavior due to different crosslinking. In various solvents, including water, buffer, and electrolyte solutions, the swelling behaviour of hydrogel was investigated and observed that TEOS amount caused less hydrogel swelling. In acidic pH, hydrogels swell the most, while they swell the least at pH 7 or higher. These hydrogels are pH-sensitive and appropriate for controlled drug release. These hydrogels demonstrated that, as the ionic concentration was increased, swelling decreased due to decreased osmotic pressure in various electrolyte solutions. The antimicrobial analysis revealed that these hydrogels are highly antibacterial against Gram-positive (*Staphylococcus aureus* and *Bacillus cereus*) and Gram negative (*Pseudomonas aeruginosa* and *Escherichia coli*) bacterial strains. The drug release mechanism was 98% in phosphate buffer saline (PBS) media at pH 7.4 in 140 min. To analyze drug release behaviour, the drug release kinetics was assessed against different mathematical models (such as zero and first order, Higuchi, Baker–Lonsdale, Hixson, and Peppas). It was found that hydrogel (CPG2) follows the Peppas model with the highest value of regression (R^2^ = 0.98509). Hence, from the results, these hydrogels could be a potential biomaterial for wound dressing in biomedical applications.

## 1. Introduction

Only a small portion of drugs spread in the body using an old drug dosage method, reaching the desired target area. The drug delivery system is used to deliver therapeutic agents, depending on the specific site and time. Various drug delivery systems are used as pharmaceutical devices, but an important research area is hydrogel drug delivery devices [1,2]. Hydrogels are adopted for drug storage and a sustained release rate is necessary for an adequate drug delivery system. Hydrogel consists of three-dimensional chains of polymers, and the nature of these polymers is hydrophilic [3]. In recent years, research on hydrogels has increased due to their vast applications in drug delivery systems, biosensors, self-healing materials, tissue engineering, and wound healing bandages [4]. Hydrogels are a combination of synthetic and natural polymers. They are cross-linked with a physical or chemical bond [5,6].

The pH-sensitive hydrogels are essential for targeted and controlled drug release at various pHs due to the differing pH of internal body fluids and organs [7]. The swelling of hydrogels is associated with ions that carry group charges. Further, it depends on many characteristics, such as the group of ions, values of pKa or pKb, degree of ionized groups, the capacity for water absorbance, amount of polymeric material, and pH of the medium. The pH and the pedant types of groups are vital to managing hydrogel characteristics [8]. Chitosan and PEI swelling were high in an acidic medium, and the reason for this was the protonation of (−NH_2_) amine groups. These are cationic hydrogels. This protonation causes repulsion and is responsible for swelling [9]. Smart hydrogels that respond to pH are among the best types of hydrogels used for sustained/directed drug release. Due to the different pH of human body liquids and internal body parts, pH-sensitive hydrogels are successful in biomedical research [10]. Biocompatible and nontoxic properties are essential for hydrogel applications in the biomedical field. An in-vivo toxicity test is used to test the cytotoxicity of hydrogels. Biocompatibility may be defined as a material that can work with a suitable host that works in a particular environment [11]. For the formation of hydrogels, synthetic and natural biopolymers are used, such as chitosan, guar gum, polyvinyl alcohol, polyethene glycol, etc. These hydrogels are used for sustained drug release [12].

Chitosan (CS) is derived from chitin, while chitin is the second most abundant polysaccharide in nature [13]. It is used to form biocompatible and biodegradable hydrogels, which are also nontoxic. CS is a material used for the formation of gels, films, and fibres. CS is widely used in the pharmaceutical, food, and cosmetic industries due to its unique antibacterial and antifungal properties [14]. These properties make CS a potential candidate in biomedical applications. However, a hydrogel with controlled drug release has been formulated using CS in drug delivery systems for wound healing in biomedical applications [15,16]. Polyvinyl alcohol (PVA) is a synthetic polymer that is hydrophilic, biocompatible, and has significant mechanical properties [17,18]. It is commonly used in biomedical applications. It is used chiefly to sustain drug systems, film formation, polymer processing, and packing [19]. Guar gum (GG) is obtained from the *Cyamopsis tetragonolobus* seeds. It is a nonionic natural polymer [20]. It is mainly used as a binding agent, stabilizer, and thickener in the pharmaceutical industry [21]. GG is famous in drug delivery systems and biomedical applications due to its remarkable biocompatibility and biodegradability [22]. GG has a gel-type nature used in the dosage to slow down the drug release, improving the drug delivery rate [23]. Recently, researchers have proven that GG is efficiently used as a drug delivery system for certain diseases [24]. Tetraethoxysilane (TEOS) is mostly used as a crosslinker [25]. It is used to help crosslink the polymers which are used as biomaterials. TEOS is new and harmless compared to earlier used cross-linkers, such as tripolyphosphate, borate, epichlorohydrin, and glutaraldehyde.

This research aimed to develop a pH-sensitive blended hydrogel film using a solution casting method for sustained drug release for wound dressing. For this purpose, a combination of synthetic (PVA) and natural polymers (CS, GG) was used, crosslinked by TEOS. This crosslinker is nontoxic and biocompatible. Different percentages of TEOS were used to develop the best hydrogel films. These films are pH sensitive and suitable for drug delivery systems.

## 2. Materials and Methods

### 2.1. Materials

Guar gum (GG), polyvinyl alcohol (PVA), Chitosan (CS), and Tetraethoxysilane (TEOS) were purchased from Merck Germany. Acetic acid_,_ calcium chloride (CaCl_2_), ethanol, Sodium chloride (NaCl), Sodium dihydrogen phosphate (Na_2_HPO_4_), Potassium chloride (KCl), and Potassium dihydrogen phosphate (KH_2_PO_4_) were purchased from Sigma Aldrich, Selangor, Malaysia. Paracetamol was obtained from WIMITS pharmaceutical Pvt. Ltd.

### 2.2. Fabrication of Hydrogel with Cross-Linker

Different formulations were designed by optimizing chitosan and guar gum composition, as shown in Table 1. Chitosan (0.5 g) was dissolved in 40 mL of acetic acid (2%) with continuous stirring for 2 h at 350–450 rpm to obtain a clear CS solution. Guar gum (0.25 g) was dissolved in 30 mL deionized water with continuous stirring for 2 h to get GG solution. PVA (0.25 g) was poured into 30 mL of deionized water and heated at 80 °C until a clear PVA solution was obtained. To obtain a homogenized mixture, CS and GG solutions were mixed with continuous stirring for one h at 55 °C. The PVA solution was then added carefully to avoid splashing into CS/GG mixture and stirred for 1 h at 55 °C. Different TEOS percentages (0.005%, 0.010%, 0.015% and 0.020%) dissolved in ethanol (5 mL) were added into the blended mixture of CS/GG/PVA dropwise and stirred for 3 h at 55 °C. These compositions were named CPG0, CPG1, CPG2, CPG3, and CPG4 according to different amounts of TEOS, as summarized in Table 1.

**Scheme 1:**
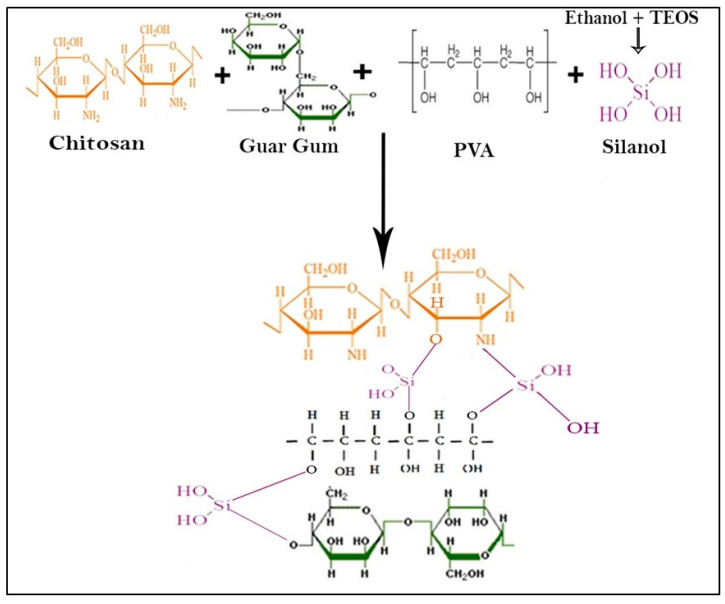
Drug loaded hydrogel.

The blended mixture was poured into the Petri dish and kept in a vacuum oven to obtain dry films of hydrogel at 55 °C. The dried hydrogel films were peeled from Petri dishes very carefully and kept in plastic bags separately for further studies. The proposed chemical mechanism is presented in Scheme 1.

CPG2 formulation was selected for drug loading and release analysis. Briefly, chitosan (0.5 g) was dissolved in 2% acetic acid (40 mL) with continuous stirring for 3 h at 350–450 rpm to obtain a clear solution. To obtain the homogenized solution, guar gum (0.25 g) was dissolved in 30 mL deionized water with continuous stirring for 2 h. To obtain a transparent PVA solution, PVA (0.25 g) was dissolved in 30 mL deionized water at 80 °C. The CS and GG solutions were mixed under continuous stirring at 55 °C for 1 h to obtain a homogenized CS/GG mixture. The PVA solution was added slowly to avoid splashing and stirred for another 1 h at 55 °C to obtain the homogenized CS/GG/PVA polymeric mixture. Paracetamol (20 mg) was dissolved into 10 mL of deionized water, added dropwise into the CS/GG/PVA polymeric mixture, and stirred for 1 h at 55 °C. TEOS (0.01%) dissolved into ethanol (5 mL) was added dropwise into the polymeric mixture and cross-linked for 3 h at 55 °C. The blended hydrogel mixture was then poured into the Petri dish and dried in an oven at 55 °C to obtain the dried hydrogel film. The drug-loaded hydrogel film was carefully preserved in a plastic bag to avoid contamination and forwarded for drug release analysis.

## 3. Characterization

Different characterizations were performed to analyze the behavior of these prepared hydrogels.

### 3.1. FTIR Analysis

The FTIR spectrum of all CPG hydrogels was obtained in ATR mode with 4 cm^−1^ of resolution and a range of 5000–500 cm^−1^ was used to determine different available functional groups.

### 3.2. SEM Analysis

Scanning electron microscopy was used to examine the morphologies of the well-dried hydrogels (SEM, JEOL-JSM-6480, Akishima, Japan). The hydrogel films were sliced and placed on a stub before being placed in the vacuum chamber to observe surface morphology.

### 3.3. Wetting Analysis

At room temperature, a VCA Optima contact angle instrument (AST Products, Inc., Billerica, MA, USA) was used to investigate the surface static contact angles of the prepared well-dried hydrogels. On the surface layer of well-dried hydrogels, 1.5 µL of distilled water was dropped.

### 3.4. Swelling Analysis

The swelling test was performed in deionized water, buffer, and electrolyte solutions. The procedure is as follows.

#### 3.4.1. Swelling in Water

Hydrogels were cut into small pieces. The weight of the dry sample was noted and it was placed in a glass Petri dish before water was added. After 10 min, the water was removed from the Petri dish and the swollen hydrogel was dried and weighed. Readings were taken after every 10 min time interval to achieve the equilibrium. For precise results, this experiment was performed in triplicate. % swelling was calculated by Equation (1).

(1) $$\mathrm{Swelling}\;(\%)=(\frac{W_{s}-W_{d}}{W_{d}})\;\times \;100$$

where ***W_s_*** = swollen weight of gel and ***W_d_*** = dry weight of gel.

#### 3.4.2. Swelling at Different pH

The hydrogel was cut into small pieces. The dry sample’s weight was noted and it was dipped in a buffer solution with different pH (4, 7 and 10). Readings were taken at the equilibrium time of each hydrogel after equilibrium was reached. The buffer solution was wasted, and the hydrogel weight was calculated. This experiment was repeated 3 times to obtain precise results.

#### 3.4.3. Swelling in Electrolytes

The hydrogel was cut into small pieces. The weight of the dry sample was noted and it was dipped in the prepared electrolyte of NaCl and CaCl_2_ of different concentrations (0.1 M, 0.5 M, and 1 M). Readings were taken at the equilibrium time of each hydrogel. After attaining the equilibrium, the electrolyte solution was wasted, and the hydrogel weight was noted again. This experiment was also repeated 3 times to obtain a precise result.

### 3.5. Degradation

Hydrogel degradation was conducted in PBS media (pH 7.4 at 37 °C) for 7 days. The well-dried hydrogels were accurately weighed (45 mg) and kept in a beaker containing PBS media to determine degradation. The hydrogels were taken out, weighted after a fixed time, and dried in an oven to put back into PBS media to determine accurate degradation. The degradation was calculated as a percentage by Equation (2).

(2) $$\mathrm{Degradation}\;(\%)=\frac{W_{i}-{\;W}_{t}}{W_{t}}\;\times \;100$$

where ***W_t_*** denotes scaffold weight at “***t***” time and ***W_i_*** is the initial scaffold weight.

### 3.6. Gel Fraction

To find the gel fraction of the prepared hydrogel, small pieces were weighed, placed in Petri dishes, and dipped in sufficient amounts of DI water for 10 h. After that, water was removed, and samples were dried in a vacuum oven at 40 °C until the weight became constant. The gel fractions were measured by Equation (3).

(3) $$\mathrm{Gel}\;\mathrm{Fraction}\;(\%)=\frac{M^{\prime }}{M{}^{\circ}}$$

where: ***M*′** = weight of the oven-dried hydrogel and ***M°*** = initial weight of the hydrogel.

### 3.7. Antimicrobial Activity

The disc diffusion method was used to study the antimicrobial activity of the CPG hydrogels against severe infection and disease-causing Gram-positive (*Staphylococcus aureus (S. aureus)* and *Bacillus cereus* (*B. cereus*)) and Gram-negative (*Pseudomonas aeruginosa (P. aeruginosa)* and *Escherichia coli* (*E. coli*)) bacteria. Antibacterial activities of all prepared hydrogels were investigated by incubating these bacterial strains at 37 °C. The sterile Petri-plates were filled with hot molten (15 mL) agar and left to solidify. The bacterial cultures were then spread properly over solidified agar using a sterile cotton swab. These hydrogels were placed over bacterial culture plates and incubated for 24 h at 37 °C. The zone of inhibition was measured in mm.

### 3.8. Drug Release

The dried drug-loaded hydrogel was dipped in 100 mL of PBS solution at 37 °C. After every 10 min, 5 mL of solution was removed with the help of a syringe and 5 mL fresh PBS was added to maintain the actual volume of the buffer. The collection of the sample was continued for 180 min. The absorbance of these samples was recorded using UV visible spectroscopy at the wavelength of 243 nm.

### 3.9. Drug release Kinetics

The drug release was evaluated in PBS media (pH 7.4 at 37 °C). The drug release kinetics was used to analyze the drug release pathway. The following mathematical models were used to evaluate release kinetics (Equations (4)–(9)) [26,27,28,29].

(4) $$\mathrm{Zero-order}:\;M_{t}{=M}_{o}{+K}_{o}t$$

(5) $$\mathrm{First}\;\mathrm{order}:\;{logC}_{o}-\frac{kt}{2.303}$$

(6) $$\mathrm{Higuchi}\;\mathrm{model}:\;{ft=Q=K}_{H}{\;\times \;t}^{1/2}$$

(7) $$\mathrm{Hixson}\;\mathrm{Crowell}\;\mathrm{model}:\;W^{1/3}-{\;W}^{1/3}=kt$$

(8) $$\mathrm{Korsmeyer-Peppas:}\mathrm{model}:\;ln\frac{M_{t}}{M_{o}}=n\;lnt+lnK$$

(9) $$\mathrm{Baker-Lonsdale}\;\mathrm{model}:\;F_{t}=\frac{2}{3}\left[1\;-{\left(1\;-\frac{M_{t}}{M_{o}}\right)}^{\frac{2}{3}}\right]\frac{M_{t}}{M_{o}}{=K(t)}^{0.5}$$

### 3.10. Statistical Analysis

The statistical analysis of the data was performed using ANOVA (post hoc test of Turkey). The collected data were calculated in triplicate (*n* = 3 and *p*-value < 0.05).

## 4. Result and Discussion

### 4.1. FT-IR Analysis

**Figure 1:**
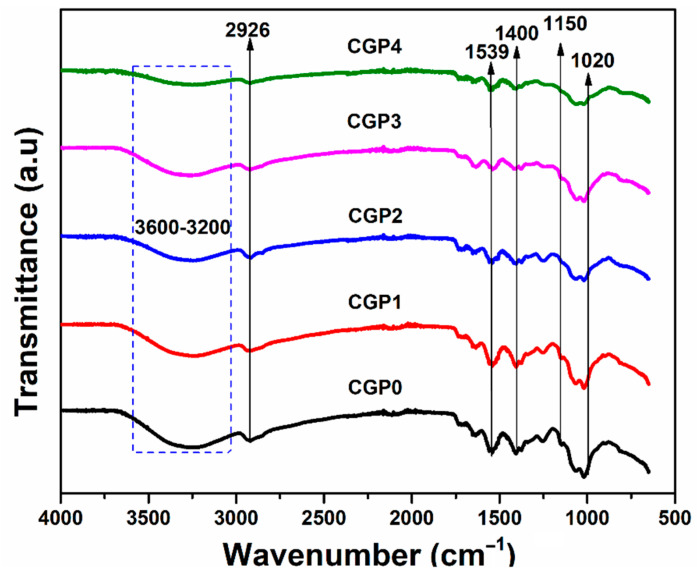
FTIR spectrum of prepared hydrogels.

FTIR results of blended hydrogel films CPG0, CPG1, CPG2, CPG3 and CPG4 are shown in Figure 1. The FTIR spectrum shows the presence of guar gum, chitosan, PVA, and TEOS. The broadband, 3000–3500 cm^−1^, shows chitosan and guar gum, corresponding to −OH/−NH_2_ molecular hydrogen bonds [30]. The absorbance peak at 1558 cm^−1^ corresponded to amide-I and amide-II groups, which confirmed the presence of chitosan. The absorbance band at 1150 cm^−1^ was conformed to a pyranose ring corresponding to chitosan and guar gum. The absorbance peak at 1750 cm^−1^ was recognized to be the non-hydrolyzed acetate group from poly (vinyl acetate) through PVA production [30]. The absorbance peak at 1020 cm^−1^ exposed the presence of Si-O-Si, which confirmed the cross-linking of TEOS with chitosan, guar gum, and PVA. Hence, all functional groups and spectral peaks confirmed the preparation of desired hydrogel formulations successfully.

### 4.2. SEM Analysis

**Figure 2:**
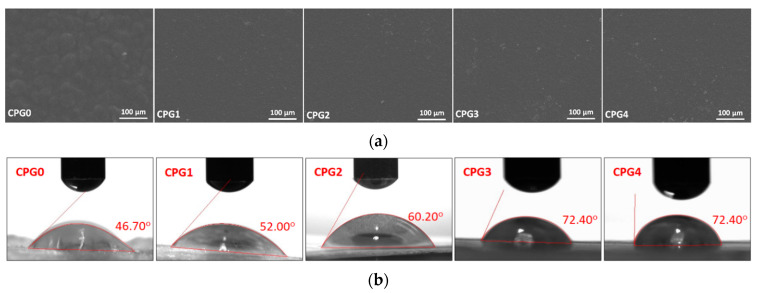
(**a**) SEM analysis of prepared hydrogels and (**b**) wetting analysis of prepared hydrogels.

The surface morphology is an essential characteristic of the wound dressing. The surface morphology of the prepared hydrogel is presented in Figure 2a. The smooth surface morphology of these hydrogels is due to the oven-drying without vacuum. It was observed that the hydrogel without cross-linker (CPG0) has a different surface morphology, i.e., rough morphology. However, the cross-linked hydrogels (CPG1, CPG2, CPG3 and CPG4) have a smooth morphology due to proper crosslinking behavior. These crosslinked hydrogels have a dense structure and demonstrate different wetting, swelling and degradation behaviors [6,31]. The hydrogel sample (CPG0) has a rough and wavy surface. It is not cross-linked, and polymers are linked randomly by forming clusters (small islands) through hydrogen bonding and other weak interactions. Hydrogels (CPG1, CPG2, CPG3, and CPG4) have a dense and smooth surface morphology due to proper crosslinking. Hence, surface morphology plays a vital role in wound healing applications.

### 4.3. Wetting Analysis

Wetting is an important characteristic of wound dressings because it indicates whether the material is hydrophilic or hydrophobic. The wetting behavior towards water was evaluated to compare the hydrogel’s surface water adsorption performance. Due to different cross-linking, water droplets were adsorbed into the hydrogel samples in different ways, which was observed for all hydrogel samples, as shown in Figure 2b. The hydrogel sample (CPG0) was found to have a lower water contact angle and exhibit hydrophilic behavior. The amount of crosslinking in the hydrogel samples (CPG1, CPG2, CPG3, and CPG4) varies. As the cross-linker increases, the wetting behavior shifts from hydrophilic to hydrophobic (i.e., hydrophilic to hydrophobic). The hydrogel sample CPG1 has the lowest contact angle (52.00°), and the hydrogel sample CPG4 has the highest water contact angle (72.40°) among all cross-linked hydrogel samples. The increased cross-linking creates a more compact and smoother surface while also increasing the structure’s close packing. As a result, as cross-linking increases, the hydrophilicity behavior shifts toward hydrophobicity. The tightly packed hydrogels prevent water absorption [7,32]. The swelling force is countered by the retractive force induced by the crosslinked polymer network in hydrogels. As a result, hydrogels that were water-insoluble during the swelling test were assumed to be crosslinked sufficiently.

### 4.4. Swelling Analysis

Hydrogels absorb water when a solvent penetrates the pore spaces between the polymeric chain network, allowing the hydrogels to expand. In some instances, external stimulators (like pH, ionic strength, and ambient temperature) can influence swelling behavior. Because of their large surface areas, hydrogels react promptly to examine swelling capacity, allowing for more fluid exchange with the environment [32]. Furthermore, higher swelling rates of hydrogels in acidic environments due to a significant increase in volume can be linked to the pH-dependent release behavior of the entrapped drug cargo. The swelling also helps to absorb extra wound exudate, which leaks blood capillaries. Wound exudate is very important for wound healing, but an excess of the wound may cause bacterial growth and need to be absorbed [33]. Hence, a hydrogel with exceptional absorbing behavior is desired for wound healing.

#### 4.4.1. Water Swelling

The swelling behavior of prepared hydrogels exhibited different swelling due to different structural and physicochemical properties. The diffusion is controlled by the attraction between polymer chains and the solvent.

##### Swelling of CS/GG/PVA Hydrogels with a Crosslinker

**Figure 3:**
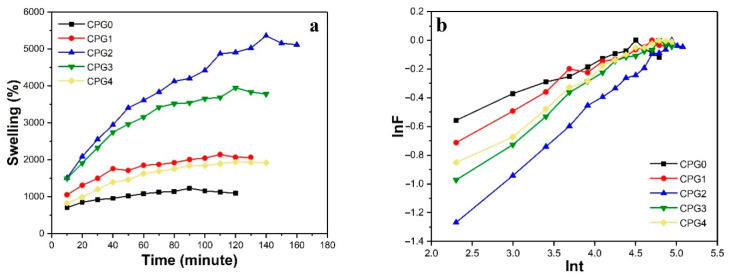
(**a**) Time vs. %swelling of CPG0, CPG1, CPG2. CPG3 and CPG4 hydrogels in DI water and (**b**) lnF vs. lnt (t) for CPG0, CPG1, CPG2. CPG3 and CPG4 hydrogels.

The behavior of CPG hydrogels with TEOS swollen in DI water with time is shown in Figure 3a. The swelling characteristics for all hydrogels were different. The swelling of hydrogel increased continuously with time intervals. The equilibrium time was different for all hydrogel samples (CPG0 = 90 min, CPG1/CPG2 = 140 min, CPG3/CPG4/CPG5 = 120 min). The first tendency to increase swelling was due to the maximum diffusion of water and pore formation. Increasing the amount of cross-linking agents increased the amount of -OH groups, which led to the bond formation between the chains of a hydrogel network, which could cause maximum swelling with a greater crosslinking agent (0.01%). The swelling study was performed in triplicate, and the results were repetitive. A maximum amount of TEOS (0.02%) of the hydrogel swelling was decreased due to increased cross-linking, which reduced the spaces between the chains of the hydrogel. As a result, water molecules could not penetrate the polymeric structure of hydrogel and swelling decreased. This tendency was consistent with the usual tendency for swelling to decrease with an increasing amount of TEOS. It can be assumed as a rule that the ability of hydrogels to swell is dependent on the degree of crosslinking [34].

##### Kinetics of Swelling

The diffusion mechanism explains the swelling behavior of hydrogels. Kinetics values of the swelling mechanism of these CPG hydrogels were calculated by Fick’s Equation (10).

(10) $${F=kt}^{n}$$

where ***F*** is the ratio between ***W_t_*** and ***W_eq_*, *k*** denotes the swelling rate constant, and ***n*** is the swelling exponent.

This equation is also called the Fick law of diffusion. These hydrogels showed that the value of *n* is less than 0.5, which means that the diffusion rate is less than the polymer relaxation rate [35,36]. A graph between lnF and lnt is shown in Figure 3b, and values of diffusion parameters are shown in Table 2.

#### 4.4.2. Swelling at Different pH Solutions

**Figure 4:**
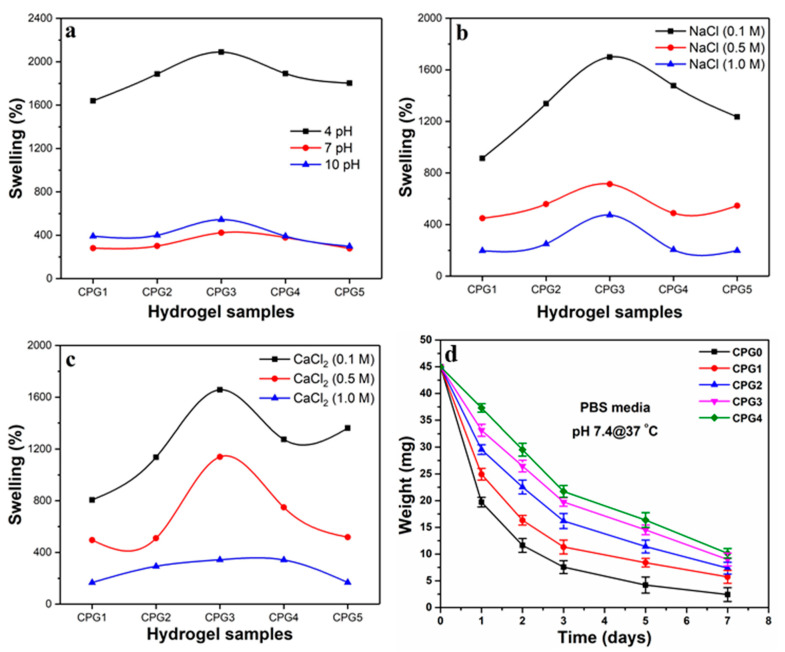
The swelling behavior of hydrogels (CPG0, CPG1, CPG2, CPG3 and CPG4) was analyzed in different media. (**a**) Swelling in aqueous media, (**b**) swelling in NaCl media, (**c**) swelling CaCl_2_ media and (**d**) degradation of hydrogels in PBS media.

Usually, the swelling in hydrogel also depends on the swelling medium. Typically, changing the pH of the solution can cause changes in the swelling of the hydrogel. To study the influence of pH in hydrogel swelling, buffer solutions of different pH (4, 7 and 10) were used. The swelling of CPG0, CPG1, CPG2, CPG3, and CPG4 hydrogels in buffer solutions of different pH is shown in Figure 4a.

These results have verified that hydrogels show the highest swelling in the acidic medium and the lowest in neutral to basic medium. The hydrogel mainly had cationic chitosan, a small amount of anionic PVA, and nonionic polymer GG in its composition. The only cationic group was responsible for swelling in a pH-dependent manner. Consequently, the protonation of the NH_2_ group results in swelling. At acidic pH, cationic groups, mainly NH^3+^_,_ were protonated and increased the number of positive charges in the polymer network, which further promoted polymer hydrophilicity. As a result, the swelling was increased [37]. The deprotonation of NH2 groups can explain the decrease in hydrogel swelling at high pH, which causes lower swelling at basic or neutral pH [38].

#### 4.4.3. Swelling in Electrolyte

Swelling is a significant phenomenon in biomedical tissue engineering and wound healing due to its resemblance with extra cells. It also controls biodegradation and sustainable drug release. The concentration of salt has an essential effect on the hydrogel swelling reaction. To study the effect of salt concentration, NaCl and CaCl_2_ were used in the experiment to check the swelling behavior. A graph was drawn between %swelling and concentration, as shown in Figure 4b,c. The results confirms that swelling was maximum in low salt concentration and minimum swelling was observed in high salt concentration. A substantial amount of salt in the solvent lowers the osmotic pressure. Consequently, diffusion is reduced, and therefore the swelling. Besides, at low ionic strength, a better swelling percentage was shown.

### 4.5. Degradation Analysis

Biodegradation is a term that describes the natural deterioration of biomaterial. However, in clinical biomaterials, biodegradation refers to the biological activities inside the body causing the biomaterial to degrade gradually. Degradation is an essential aspect to recognize when using biomaterials for medical purposes because the period they should be kept in the body determines their capacity to perform for a particular application. The adaptability of polymeric materials allows specific biodegradable hydrogels to control drug release and enhance cell growth. Biodegradable hydrogels for wound healing with sustained drug release have the potential to avoid a chronic immune reaction, as well as to avoid removal surgery [39]. The degradation analysis of hydrogels was performed to determine their degradability. The hydrogel CPG0 has maximum and the hydrogel CPG4 has minimum degradation. It can be seen from Figure 4d that hydrogel CPG0 has fast degradation, hydrogel CPG4 has slow degradation and hydrogel CPG2 has sustained and controlled degradation. The degradation may be due to available weak (van der Waal and hydrogen bonding etc.) and strong (glycosidic and covalent bonding, etc.) interactions. Due to the degrading nature of hydrogels interacting with PBS media at 37 °C and 7.4 pH, these interactions weakened. It was observed that increasing crosslinking has an inverse effect on degradation [6]. Hence, crosslinking is a vital parameter to optimize degradation, to support controlled and sustained drug release.

### 4.6. Gel Fraction

**Figure 5:**
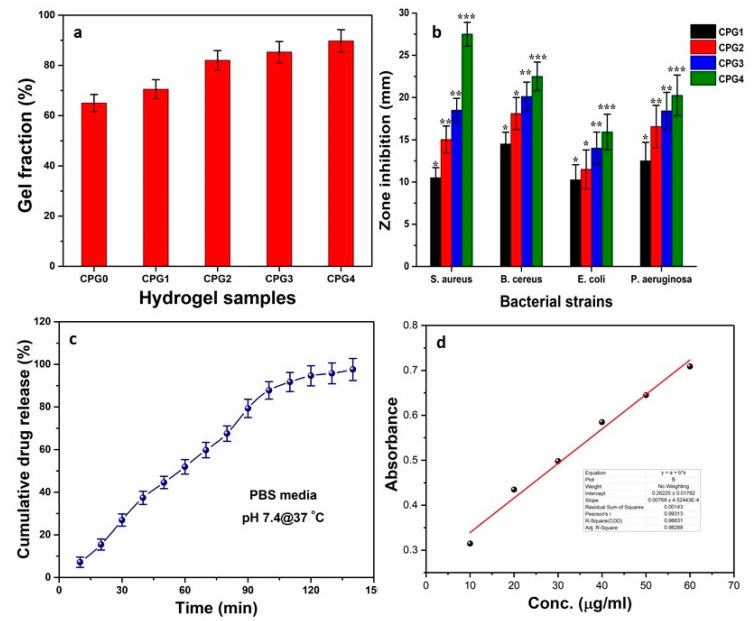
(**a**) Percentage of gel fraction (**b**) zone inhabitation against Gram positive (*S.* and *B. cereus*) and Gram negative (*P. aeruginosa* and *E. coli*) bacterial strains, (**c**) cumulative release (%) of paracetamol from CPG2, (**d**) standard curve of paracetamol at 243 nm. (* *p* < 0.05, ** *p* < 0.01 and *** *p* < 0.001 and *n* = 3.)

Gel fraction (gelation or gel transition) refers to the formation of a gel from a polymeric system. Polymers can form links between their chains due to various functional groups, and a linking reaction progresses between the polymers at a certain point to form a gel. A single macroscopic molecule is formed due to polymeric interaction, which is known as the gelation point. The polymeric system loses its fluidity with enhanced viscosity [40]. Gelation can be caused by a physical (H-bonding, hydrophobic, electrostatic, and π–π interactions) or chemical covalent interaction. These physicochemical interactions play an essential role in gel formation. Quick gelation is very important in injuries to stop bleeding and wound healing. It absorbs wound exudate to hinder antibacterial activities [41,42]. The gel fraction of cross-linked determines the degree of cross-linking. The percentage of gel fraction against hydrogels CPG0, CPG1, CPG2, CPG3 and CPG4 was observed as 65.01%, 70.51%, 82.02%, 85.32%, 89.72%, respectively as shown in Figure 5a. These results confirm that increasing the crosslinker amount increases the gel fractions of hydrogels. The increasing gel fraction may be due to the increased physicochemical interaction of polymers and TEOS, as well as covalent crosslinking due to the increased TEOS amount.

### 4.7. Antibacterial Activity

Skincare dressings support wound healing by releasing antibacterial agents, hastening wound closure and reporting changes in the condition of the wound. The uncontrolled administration of antibacterial agents, such as drugs, peptides, or nanoparticles, remains challenging. Furthermore, bacterial resistance to antibiotics drives the development of new antibacterial wound dressings. Because of their ability to effectively inhibit bacterial infections, antibacterial hydrogels are recognized as essential biomaterials for wound healing applications [43]. Currently, hydrogels with an antibacterial function are the main focus in biomedical research. Hence, we are reporting economically and physically blended hydrogels with enhanced antibacterial activity, as shown in Figure 5b. The results have shown that these hydrogels present good activity against tested bacteria, so these hydrogels are biocompatible. The exceptional antibacterial activity of CPG hydrogels was due to the cationic nature of CS, which inhibits negatively charged residual growth in the cell wall of bacteria. The negatively charged lipopolysaccharide groups connect with protonated ammonium groups of CS. The charge interaction distracts the cell membrane’s osmotic balance, leading to the bacteria’s cell lysis [44,45]. These hydrogels interact with lipophilic components, like lipoproteins, liposaccharides, and phospholipids, with a bacterial membrane via electrostatic and hydrophobic means. As a result, bacteria adsorb and immobilize on the hydrogel surface. The hydrogel may enter the cell via the cell membrane to bind bacterial ribosomes, disrupting protein transcription and inhibiting bacterial activity. Since increasing TEOS amounts cause an increase in the water contact angle due to close packing of the polymeric matrix, that shifts more hydrophilic behaviour to become hydrophobic [6]. An increasing water contact angle also discourages bacterial adherence, which may be why CPG4 exhibited more antibacterial activities against all bacterial strains.

### 4.8. Drug Released Analysis

Drug release analysis was performed in PBS at 7.4 pH. The graph between % cumulative release and time is shown in Figure 5c. The results have shown 98% drug release in 140-min time intervals. The drug-release mechanism is always due to diffusion, whereby drug molecules come out of the polymeric network depending on the hydrogel’s porosity [46]. Paracetamol release shows sustained drug release in CPG2 so that this hydrogel can be suitable for controlled drug delivery applications due to optimized physicochemical behavior [47]. The absorbance of standard solutions with known concentrations was measured by a UV-visible spectrometer and plotted between absorbance and concentration. The calibration curve determines the cumulative drug release from the drug-loaded hydrogel. The calibration of paracetamol is shown in Figure 5d.

### 4.9. Drug Release Kinetics

**Figure 6:**
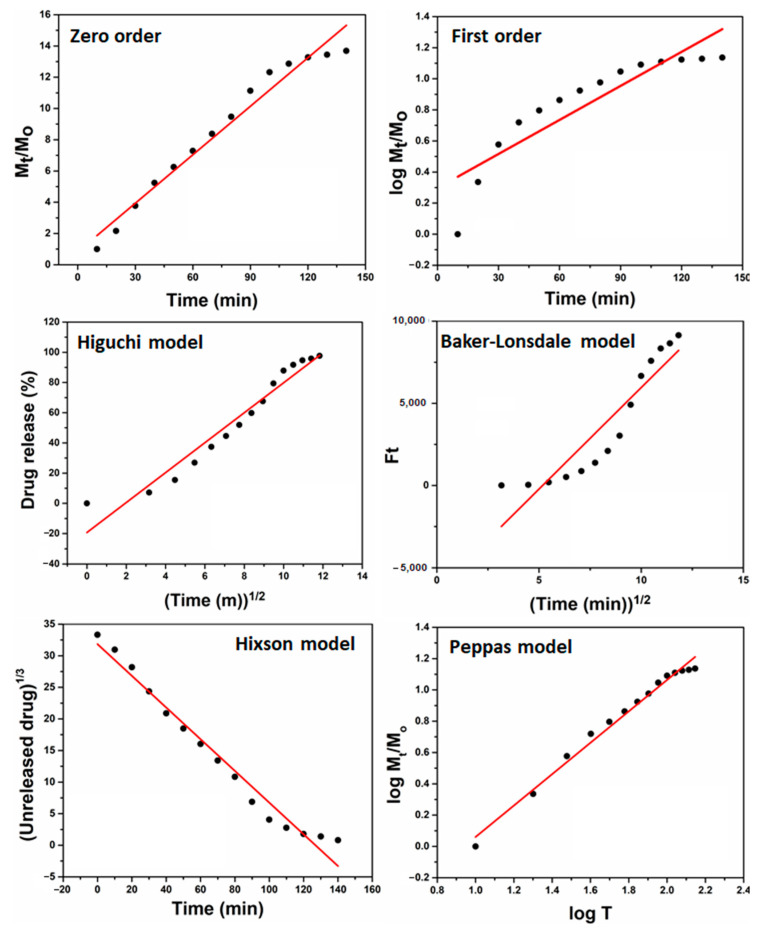
Drug release kinetics of prepared hydrogel with different mathematical models (Zero-order, First-order, Hixson, Baker-Lonsdale, Higuchi and Peppas model to analyze drug release behavior.

The drug release kinetics were evaluated using mathematical models (Equations (4)–(9)) to understand drug release behavior, as presented in Figure 6. The polymeric matrix of hydrogel supports sustained and controlled drug release. The degradation and swelling affect drug release kinetics, and curve fitting was studied by different fitting models (such as zero and first order, Higuchi, Baker–Lonsdale, Hixson, and Peppas). The drug-loaded hydrogel has shown the different kinetic release behavior of paracetamol, and the regression coefficient (R^2^) is presented with other parameters in Table 3. The paracetamol loaded hydrogel follows the Peppas model due to the highest value of regression coefficient (R^2^ = 0.98509), and it is also called the “Power-law,” confirming the drug release from the polymeric system. Under water diffusion, swelling, and dissolution of the polymeric matrix, the power-law explains the drug release mechanism [48]. Hence, the drug release kinetics follows the Peppas model, confirming the drug release from the polymeric system.

## 5. Conclusions

We have reported the preparation of hydrogels by the physical blending of polymer with PVA, crosslinked with TEOS. FTIR analysis has confirmed the successful preparation of hydrogels and SEM morphologies demonstrate different morphological behavior. The wetting analysis confirms an increase in water contact angle with increasing TEOS amount, and the more hydrophilic shifts towards less hydrophilic behavior. The hydrogel sample CPG3has exhibited a maximum swelling of 2159% after 140 min in water. The hydrogel CPG2was further used for drug release analysis. The maximum swelling (%) was obtained at pH 4 and the minimum at neutral. Decreased swelling was observed with an increased concentration of electrolytes of these hydrogels, and it is also noted that increasing the amount of TEOS decreased degradation. Antimicrobial activities showed that these hydrogels are highly antibacterial against Gram positive and Gram-negative bacterial strains. A sustained drug release was found in PBS media, and 98% paracetamol release was observed in 140 min following the Peppas drug release model. Hence, the prepared hydrogels are pH-sensitive with sustained drug release. Indeed, these hydrogels demonstrate potential for biomedical applications in wound healing and tissue engineering. Studies of these hydrogels are ongoing to further explore their properties.

## Figures and Tables

**Table 1:** The formulation used in the preparation of CPG hydrogels.

Sample	Nomenclature	Chitosan (g)	Guar Gum (g)	PVA (g)	TEOS (%)
1	CPG0	0.5	0.25	0.25	0
2	CPG1	0.5	0.25	0.25	0.005
3	CPG2	0.5	0.25	0.25	0.010
4	CPG3	0.5	0.25	0.25	0.015
5	CPG4	0.5	0.25	0.25	0.020

**Table 2:** Swelling kinetics parameters of CPG hydrogels.

Parameters	CPG0	CPG1	CPG2	CPG3	CPG4
*n*	0.20103	0.2628	0.459	0.371	0.344
**Intercept**	−0.98218 ± 0.011	−1.28157 ± 0.12	−2.30061 ± 0.23	−1.78842 ± 0.13	−1.63906 ± 0.12
*K*	0.3745 ± 0.001	0.278 ± 0.002	0.1002 ± 0.0012	0.1672 ± 0.0014	0.1942 ± 0.0012
**Regression**	0.95751 ± 0.012	0.98232 ± 0.014	0.995 ± 0.011	0.988 ± 0.021	0.989 ± 0.022

**Table 3:** Drug release kinetic by different models.

Models	Intercept	Slope	R^2^
**Zero order**	0.83045 ± 0.47	0.10348 ± 0.31	0.96641 ± 0.19
**First order**	0.29692 ± 0.28	0.0073 ± 0.11	0.79207 ± 0.13
**Higuchi**	−19.28981 ± 0.31	9.91029 ± 0.22	0.94397 ± 0.21
**Baker-Lonsdale**	−6399.68312 ± 0.28	1235.80484 ± 0.10	0.84256 ± 0.13
**Hixson**	31.83667 ± 0.12	−0.25085 ± 0.12	0.97203 ± 0.17
**Peppas**	−0.94357 ± 0.11	1.00373 ± 0.20	0.98509 ± 0.14

## Data Availability

Data contained within the article.
